# The secondary resistome of multidrug-resistant *Klebsiella pneumoniae*

**DOI:** 10.1038/srep42483

**Published:** 2017-02-15

**Authors:** Bimal Jana, Amy K. Cain, William T. Doerrler, Christine J. Boinett, Maria C. Fookes, Julian Parkhill, Luca Guardabassi

**Affiliations:** 1 Department of Veterinary Disease Biology, Faculty of Health and Medical Sciences, University of Copenhagen, Frederiksberg, Denmark; 2 Department of Biomedical Sciences, Ross University School of Veterinary Medicine, Basseterre, St Kitts, West Indies; 3 Pathogen Genomics, Wellcome Trust Sanger Institute, Cambridge, UK; 4 Department of Biological Sciences, Louisiana State University, Baton Rouge, LA, USA

## Abstract

*Klebsiella pneumoniae* causes severe lung and bloodstream infections that are difficult to treat due to multidrug resistance. We hypothesized that antimicrobial resistance can be reversed by targeting chromosomal non-essential genes that are not responsible for acquired resistance but essential for resistant bacteria under therapeutic concentrations of antimicrobials. Conditional essentiality of individual genes to antimicrobial resistance was evaluated in an epidemic multidrug-resistant clone of *K. pneumoniae* (ST258). We constructed a high-density transposon mutant library of >430,000 unique Tn*5* insertions and measured mutant depletion upon exposure to three clinically relevant antimicrobials (colistin, imipenem or ciprofloxacin) by Transposon Directed Insertion-site Sequencing (TraDIS). Using this high-throughput approach, we defined three sets of chromosomal non-essential genes essential for growth during exposure to colistin (n = 35), imipenem (n = 1) or ciprofloxacin (n = 1) in addition to known resistance determinants, collectively termed the “secondary resistome”. As proof of principle, we demonstrated that inactivation of a non-essential gene not previously found linked to colistin resistance (*dedA*) restored colistin susceptibility by reducing the minimum inhibitory concentration from 8 to 0.5 μg/ml, 4-fold below the susceptibility breakpoint (S ≤ 2 μg/ml). This finding suggests that the secondary resistome is a potential target for developing antimicrobial “helper” drugs that restore the efficacy of existing antimicrobials.

Antimicrobial resistance is an emerging global healthcare crisis with significant impact on human health and economy^1^. This crisis is worsened by the dearth of new antimicrobials, especially against Gram-negative pathogens, and by the spread of high-risk multidrug-resistant (MDR) clones. Of particular concern is the emergence of MDR *Klebsiella pneumoniae* strains resistant to carbapenems, a class of last resort antibiotics for treatment of severe Gram-negative infections^2^. Carbapenem resistance is mainly associated with the plasmid-borne *bla*_KPC_ gene encoding carbapenem-hydrolyzing *K. pneumoniae* carbapenemase (KPC)^3^. In addition to *bla*_KPC_, MDR *K. pneumoniae* isolates carry an average of 11–13 acquired resistance genes, which confer resistance to virtually all antimicrobial agents available in clinical practice^4^. The emergence of carbapenem resistance has forced the reintroduction into clinical practice of colistin (CST), an antimicrobial peptide with known nephro- and neurotoxic effects^5^. Unfortunately, MDR KPC-producing *K. pneumoniae* such as the epidemic clone sequence type 258 (ST258) have developed CST resistance largely by acquiring mutations in the chromosomal LPS-modification regulatory genes, which reduces negative charge on the bacterial surface and consequently affinity towards positively charged peptides^6^. To date, no genome-wide antimicrobial drug-gene interaction studies have been performed to evaluate the contribution of individual genes to the resistance phenotype in this high-risk MDR clone.

The recent advances in next-generation sequencing combined with transposon mutagenesis has allowed development of novel tools to study gene-phenotype connections at the genome-scale and in a high-throughput manner^7^,^8^. Such tools evaluate the involvement of each gene simultaneously in a particular condition by measuring differences in transposon insertion abundance after applying selection to a saturated mutant library, compared to a control. One of these tools, Transposon Directed Insertion-site Sequencing (TraDIS), has been used to identify essential genes, screen for virulence factors and genes essential for specific biological processes (e.g. sporulation), as well as novel targets for antimicrobial development^9^,^10^,^11^. This method provides a systems-level view of the interdependent gene networks that respond to stress, for example antimicrobial stress^12^. The usefulness and success of this method depend on the availability of saturated transposon insertion mutant libraries, which in turn rely on the transformation or transposition efficiency of the strain used for constructing the library.

In this study, we constructed the first saturated transposon mutant library of MDR KPC-producing *K. pneumoniae* ST258 and, for the first time in *Klebsiella pneumoniae,* we employed TraDIS. We evaluated the contribution of each individual gene to resistance to three clinically important drugs which this strain is resistant to: colistin (CST; a polymyxin), imipenem (IPM; a carbapenem), and ciprofloxacin (CIP; a fluoroquinolone). TraDIS unveiled the existence of chromosomal non-essential genes that become essential in presence of therapeutic concentrations of antimicrobials. Considering that known antimicrobial resistance elements can be regarded as primary resistance genes, we designated these genes individually as “Secondary Resistance Genes” (SRGs) and collectively as the “Secondary Resistome” (SR) for each antimicrobial. As proof of principle, we demonstrated that inactivation of a SRG restored susceptibility to CST.

## Results

### MDR *K. pneumoniae* ST258 genome sequence

*K. pneumoniae* ST258 strain RH201207 (Table S1) is a clinical isolate obtained from Public Health England in 2012. RH201207 is resistant to CST, IPM and CIP with Minimal Inhibitory Concentrations (MICs) of 8, 16 and 32 μg/ml, respectively. In addition, the strain displays resistance to aminoglycosides, chloramphenicol, macrolides, sulphonamides and trimethoprim. For this study, whole genome sequencing (WGS) of RH201207 was performed to develop the reference genome required for TraDIS analysis using a combination of PacBio and Illumina platforms, and annotated sequences were submitted in European Nucleotide Archive (ENA) (accession numbers LT216436-LT216440). WGS identified 5.4 Mb of chromosomal sequence with 5798 genes, including 89 and 25 tRNA and rRNA encoding genes, respectively, an average GC content of 58.52% and four extrachromosomal plasmids with the size of 18, 42, 117 and 214 kb. Genome analysis revealed the presence of multiple resistance genes in the plasmid and chromosomal mutations conferring resistance to: β-lactams (*bla*_TEM_, *bla*_SHV_, *bla*_OXA-9_, and *bla*_KPC-3_), aminoglycosides (*aac6′* and *aph3*), chloramphenicol (*catA*), macrolides (*mphA*), polymyxins (mutations in *phoQ* and an IS insertion in *mgrB*), quinolones (*oqxAB*), sulphonamides (*sul1*), and trimethoprim (*dhfrA*).

### Construction of a high-density mutant library and TraDIS sequencing

**Figure 1:**
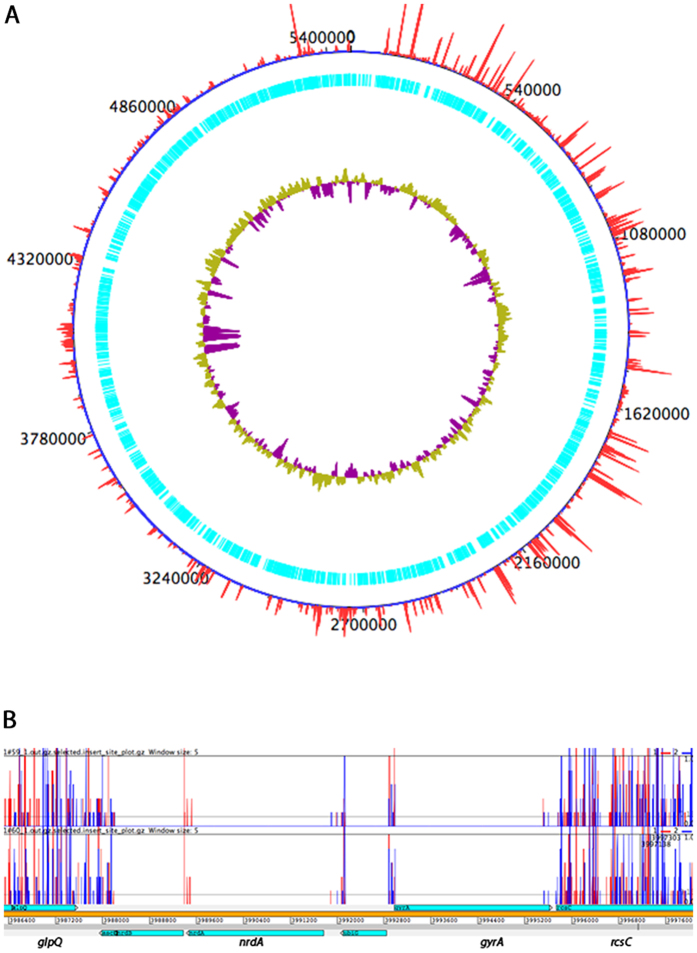
(**A**) Mapping of transposon insertion sites to the genome of *Klebsiella pneumoniae* ST258 RH201207. The chromosomal sequence is shown with open reading frames in cyan and the base positions are given in black. The inner ring represents the GC content (purple indicates values below average, whereas yellow-green indicates values above average). The red lines on the outer ring represent the numbers of Tn*5* insertions at each position in the genome, which varies from 1 to 631. (**B**) Chromosomal section of the Transposon Directed Insertion-site Sequencing (TraDIS) insertion map of *Klebsiella pneumoniae* ST258. DNA was extracted from mutant library and TraDIS analysis was performed in duplicate, subsequently insertion site reads were plotted on the chromosomal sequence using Artemis. The figure shows that transposon insertions occurred in non-essential genes (*glpQ* and *rcsC*) but not in essential genes (*nrdA* and *gyrA*).

A RH201207 mutant library of over 1 million individual mutants was constructed using modified Tn*5* transposon^13^. Since the target strain was susceptible to tetracycline, a Tet^R^ resistance cassette was PCR amplified using Tn*5* mosaic element tagged primers. The amplified cassette was incubated with Tn*5* transposase to form an *in vitro* transposome complex, which was electroporated into competent RH201207, followed by mutant selection on agar supplemented with tetracycline. During competent cell preparation, growth media was supplemented with EDTA to increase the transformation efficiency by destabilizing the cell envelope^14^. Over 1 million colonies, each representing an individual mutant, were collected from multiple batches of electroporation and pooled. Saturation of transposon insertions over the ST258 genome was confirmed by TraDIS analysis^15^. Briefly, 32 million single ended 50 bp Illumina sequence reads of insertion sites were generated and mapped onto the full genome sequence of RH201207, leading to identification of more than 431,000 unique transposon insertions distributed across the genome (Fig. 1A) with an average of one transposon insertion in every 19 nucleotides. Complete absence of insertions in genes previously known to be essential in *K. pneumoniae*, such as *gyrA* and *nrdA*^16^,^17^ (Fig. 1B), demonstrated the technical success of each step, from library construction to mapping of TraDIS insertion site reads over the genome (Table S3). Analysis of the unchallenged TraDIS library DNA showed that from 5798 genes, 642 genes were determined to be essential (11% of the genome) and 49 ambiguous, that is, not essential or non-essential, using the parameters defined in Dembek *et al*. 10.

### Identification of non-essential genes important for growth with antimicrobials

The contribution of each non-essential gene to antimicrobial resistance was evaluated by measuring depletion of transposon insertion mutants from the population following exposure to CIP, CST and IPM. To mimic therapeutic levels, we chose a concentration (2 μg/ml) that is 2 fold lower than the resistance breakpoint for these drugs (R ≥ 4 μg/ml)^18^,^19^. TraDIS analysis was performed and the insertion density was compared for each gene between antimicrobial-exposed and unexposed (control) cultures. Genes with a significant mutant depletion during antimicrobial exposure, as defined by a 4 fold or higher decrease in the number of insertion sites, were tabulated. In addition to known primary antimicrobial resistance genes and associated regulatory elements, namely *oqxAB* for CIP^20^, the *arn/pmr* operons for CST^6^ and *bla*_KPC3_ for IPM^21^, we identified 35 non-essential chromosomal genes/operons involved in CST resistance (Table 1), and single chromosomal genes involved in resistance to CIP and IPM (Table 2).

A gene (*dedA*) encoding putative integral membrane protein (DedA) not previously known to be associated with CST resistance, displayed the highest mutant depletion within the library upon exposure to CST. Insertions detected in this SRG decreased by 512 fold in the presence of CST. The functional classification of the remaining 34 CST SRGs identified by this study were membrane biogenesis (*wabN, galE, galU, fabR, pgi, bamB, mrcB*)^22^,^23^,^24^,^25^,^26^, putative ECA (enterobacterial common antigen) polymerase (*RH201207_01572*), maintenance of outer membrane integrity (*pal, tolQRAB*)^27^, cell division (*dedD, envC*)^28^, presentation of virulence factors (*galU*)^29^,^30^, transcription regulation (*cpxR, hupA*)^31^, membrane chaperone/protease (*fkpA, degP*), and energy metabolism (*sdhD, yhcB, fre*)^32^,^33^ (Table 1). As expected, each gene in the *arn* operon, which is needed to confer CST resistance via modification of Lipid A, had a decreased insertion count in the presence of CST. In particular, inactive mutants in *arnT* and *arnE* were depleted by 256 fold in CST-exposed samples.

Interestingly, *nhaA* and *ydiE* were the only chromosomal genes identified in the SR of IPM and CIP, respectively (Table 2). Therapeutic levels of IPM in the culture medium decreased insertion abundance by 4 fold for *nhaA*, which encodes a Na^+^/H^+^ antiporter^34^. Although *ydiE* displayed a 4-fold mutant depletion following exposure to CIP, the q value was not significant (>0.01). For this reason, this gene was not selected to perform additional experiments.

### Effect of CST concentration on depletion of insertion mutants

**Figure 2:**
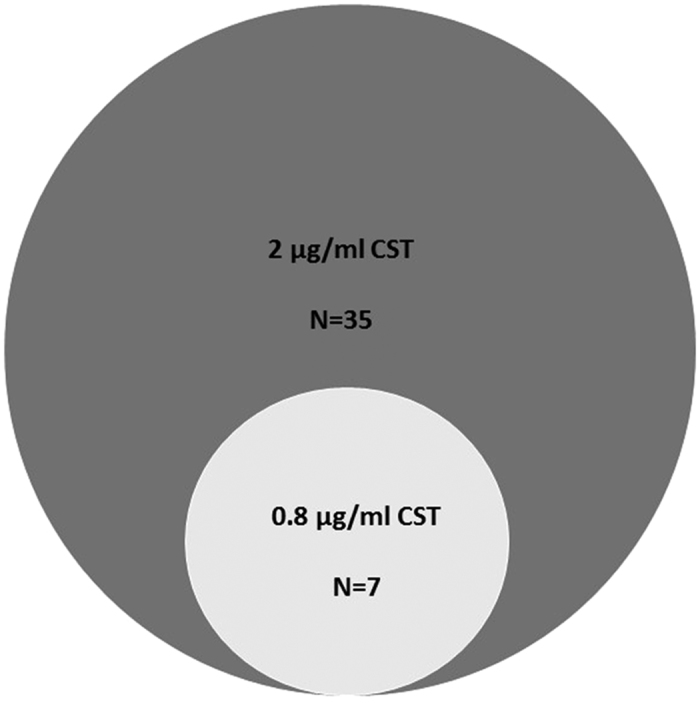
Venn diagram of the colistin (CST) secondary resistance genes (SRGs) detected in Klebsiella pneumoniae ST258 upon exposure to different drug concentrations. The diagram shows the overlap between the SRGs detected by Transposon Directed Insertion-site Sequencing (TraDIS) analysis after exposure of the *K. pneumoniae* ST258 mutant library to 2 μg/ml (dark grey circle) and 0.8 μg/ml (light grey circle) of CST, respectively.

**Figure 3:**
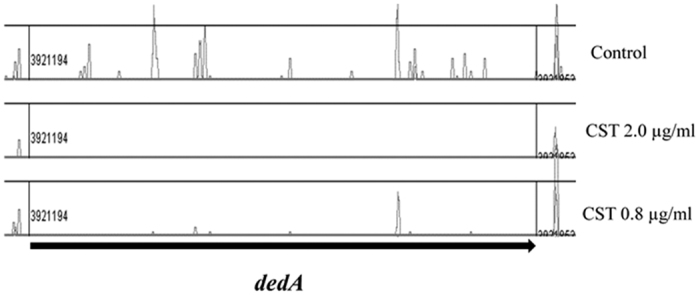
Transposon insertion abundance in dedA at different colistin (CST) concentrations. Transposon insertions detected in *dedA* by Transposon Directed Insertion-site Sequencing (TraDIS) analysis after culture of the *Klebsiella pneumoniae* ST258 mutant library in the presence of 2 or 0.8 μg/ml of CST and in the absence of antibiotic (control).

To understand the dynamics of the mutant population at different CST concentrations, the library was also exposed to a lower concentration (0.8 μg/ml), which corresponds to 1/10 of the MIC of CST in RH201207. Exposure to this drug concentration reduced the size of the CST SR from 35 to 7 SRGs (Table 1, Fig. 2), as well as the level of depletion of insertions for the 6 SRGs that were also identified at the higher concentration. Insertions in the primary resistance operon (*arnA-T*) showed equivalent levels of depletion at high confidence at both CST concentrations. Similar to the *arn* operon, one SRG, *RH201207_00408*, encoding a putative glycosyltransferase involved in cell wall biosynthesis, was found with decreased insertions at both concentrations (Table 1). The other 6 genes that were found important at both concentrations are functionally involved in membrane biogenesis (*dedA, wabN, RH201207_00413, galU* and *pgi*) or maintenance of envelope integrity (*pal*). At the lower CST concentration, the SRG *dedA* had a less dramatic reduction in insertions, compared to the control, than the higher concentration (Fig. 3). Interestingly, four genes (*galE, lpp, surA* and *RH201207_02818*) involved in membrane biogenesis were implicated in resistance at the higher concentration but not at the lower one.

### Confirmation of selected SRGs involvement in antimicrobial resistance

The two genes *dedA* and *nhaA,* which were identified by TraDIS analysis as the SRGs with the largest reduction in insertion levels during CST and IPM exposure, respectively, were each knocked out individually by Lambda Red recombination^35^,^36^. While no reduction in the MIC of IPM was observed when *nhaA* was deleted, targeted deletion of *dedA* rendered RH201207 susceptible to CST as evidenced by a 16-fold reduction of the MIC from 8 to 0.5 μg/ml. Sequencing of the *dedA* mutant confirmed successful removal of *dedA* and no secondary mutations (Fig. S1). This *dedA* mutant was also successfully complemented via induction of DedA from arabinose inducible promoter of a synthetic plasmid (pBAD) carrying *dedA*, which restored CST resistance in the deletion mutant (Fig. S2). Deletion of *dedA* did not reduce the fitness of strain, as the growth rate of the mutant matches the wild type in drug-free medium (Fig. S3). Further, the effect of removing *dedA* was drug-specific as the mutant did not display altered MIC for any other antimicrobials tested, beyond CST (data not shown). To test the possibility of spontaneous reversion of CST resistance via suppressor mutations, MIC plates were incubated for multiple days, and no growth was observed after additional incubation.

### DedA homology to human and bacterial proteins

*In silico* analysis using NCBI-BLAST tools (http://blast.ncbi.nlm.nih.gov) did not find any homology of DedA from *K. pneumoniae* to human protein. Proteins homologous to *dedA* from other Enterobacteriaceae, including *Salmonella enterica, Shigella flexneri, Citrobacter freundii,* and *Escherichia coli* were identified as having high amino acid sequence identity. Proteins with ≥90% sequence identity were identified as hypothetical DedA family proteins and displayed ≥94% query coverage with minimum E value of 2e-144, indicating that dedA is conserved across Enterobacteriaceae.

## Discussion

This study reveals a complete set of SRGs that are implicated in resistance to three clinically relevant antimicrobials that, excluding known resistance genes, collectively make up the SR of MDR *K. pneumoniae* ST258. Although these genes were not previously known to be associated with antimicrobial resistance, their functionality was demonstrated to be important only when this high-risk clone was cultured in the presence of antimicrobials. Conditional gene essentiality was first assessed based on significant (≥4 fold) decreases in the number of transposon insertions observed during antimicrobial exposure, as measured by TraDIS. This approach was shown to be valid for identification of SRGs, since insertions in the known primary CST resistance determinant (*arn* operon) were drastically reduced after exposure to CST. Using TraDIS, the contribution of each chromosomal non-essential gene to any given phenotype can be determined in a high-throughput manner by combining large-scale mutagenesis and next-generation sequencing. This approach is less labour intensive compared to signature-tagged mutagenesis, which has been used extensively to identify virulence genes in *K. pneumoniae*^37^.

The size of the CST SR decreased when the library was exposed to a lower concentration of CST, suggesting a concentration-dependent involvement of the cellular metabolic processes, where resistance mechanisms seem to become more complex at higher levels of CST. It has been previously shown that gene expression profiles of *Acinetobacter baumannii* are substantially altered depending on the exposure dose of polymyxins^38^. The SRGs that were detected at both high and low concentrations are functionally involved in membrane biogenesis and maintenance of envelope integrity, whereas those only detected at high concentration are related to the core processes of transcription regulation, stress response, cell division and energy metabolism, in addition to membrane biogenesis (Table 1). These results indicate the key importance of the membrane biogenesis and maintenance machinery for CST resistance.

We demonstrated that CST susceptibility is restored by inactivation of the SRG *dedA*, which was identified by TraDIS analysis as the gene with the highest decrease in insertions during CST exposure. Targeted inactivation of *dedA* restored CST susceptibility by reducing the MIC of the wild type strain from 8 μg/ml to the 0.5 μg/ml. This is well below the susceptibility breakpoints (S ≤ 2), as defined by the European Committee on Antimicrobial Susceptibility Testing (EUCAST) for Enterobacteriaceae^19^ and by the Clinical and Laboratory Standards Institute (CLSI) for other Gram-negative pathogens such as *Pseudomonas aeruginosa* and *Acinetobacter* spp^18^. This is a proof of principle that CST resistance can be reversed by targeting a non-essential gene that until today has not been linked to this antibiotic resistance. Such a proof of principle is highly clinically relevant since CST is the last resort drug for treatment of KPC-producing MDR *K. pneumoniae*. Moreover, ST258 is the most commonly reported KPC-producing clone associated with colistin resistance^39^,^40^,^41^.

The SRG *dedA* had several features that make it an attractive CST “helper” drug target candidate. No homology is observed in human proteins, indicating that is safe for use. It is highly conserved across other Enterobacteriacae, indicating that is has the potential to be important in conferring resistance across multiple bacteria and that its function is non-redundant in *K. pneumoniae* ST258. The consistent drop in MIC of knockout mutants throughout multiple biological replicate experiments supports a low likelihood of the development of a spontaneous suppressor mutation of Δ*dedA*. If a suppressor mutation developed easily, it would have likely emerged during exposure of the library to antimicrobials, during the construction of deletion mutants or in the culturing step that was used for MIC determination. Furthermore, no additional growth was observed when MIC testing plates of the Δ*dedA* mutant were incubated for multiple days, supporting that DedA plays a specific role that cannot be substituted by altering the genome or metabolic network in the studied strain. This is an important characteristic that should be considered for any protein as a target for development of innovative antimicrobial “helper” drugs able to restore CST susceptibility. Moreover, the lack of fitness cost associated with *dedA* deletion (Fig. S3) suggests that potential helper drugs targeting DedA would have no or very limited antimicrobial activity, which is another desirable feature for a helper drug, as it minimizes the risk to select helper drug-resistant strains. However, the potential use of DedA as a helper drug target needs to be further validated by confirming the effects of *dedA* deletion on colistin susceptibility in multiple CST-resistant *K. pneumoniae* strains with genetic backgrounds other than ST258 and with primary CST resistance determinants other than the one observed in this clone (i.e. *phoQ* mutation and an IS insertion in *mgrB*). In addition, *in vitro* suppressor mutation frequency studies as well as *in vivo* animal experiments evaluating clinical efficacy of CST in animals challenged with Δ*dedA* mutant are warranted for comprehensive validation of this potential drug target.

To the best of our knowledge, this is the first study identifying the contribution of DedA to CST resistance in *K. pneumoniae.* This result supports the usefulness of TraDIS to identify novel SRGs that have not been previously known to be associated with antimicrobial resistance. The *dedA* gene has never been studied in *K. pneumoniae*, but inactivation of its homologues *yqjA* and *yghB* in *E. coli* has been shown to result in temperature-sensitivity and defects in growth^42^,^43^. Recent studies in *E. coli* have shown that DedA homologue YqjA is an inner membrane protein with four putative transmembrane domains, which enable growth at high pH and may have roles in drug efflux using energy of the membrane proton gradient^44^,^45^,^46^. YqjA has also been found involved in resistance to the antimicrobial peptide magainin in *Salmonella enterica*^47^. A *dedA* family gene has been implicated in resistance to antimicrobial peptides in *Neisseria meningitidis*^48^. Based on the current knowledge regarding the function of DedA-homologs in other species, DedA could be involved in *K. pneumoniae* membrane biogenesis, lipid A modification, membrane integrity or efflux. Our finding that *dedA* deletion specifically restored CST sensitivity in the epidemic clone ST258 without changing the MICs of other antimicrobials suggests that the effect of this mutation is antibiotic-specific. Further research is needed to elucidate the exact function of this protein in *K. pneumoniae*, its possible involvement in the CST resistance machinery of *E. coli* and other Gram-negative bacteria, and the molecular mechanism of colistin potentiation upon inactivation of *dedA*.

The limited SR identified by TraDIS for IPM and CIP suggests that the IPM and CIP resistance phenotypes in MDR *K. pneumoniae* ST258, which are conferred by KPC-3 carbapemenase and OqxAB efflux pump, respectively, do not require significant contribution by SRGs at the concentration tested in this study (2 μg/ml). Notably, this concentration is well below the MICs of IPM and CIP in this strain (16 and 32 μg/ml, respectively) and thus may not be sufficient to induce the level of cellular stress required to engage the full SR for these antimicrobials. The high MIC of IPM may also explain why the MIC did not change upon *nhaA* inactivation, despite the 4-fold depletion of insertions that was observed by TraDIS analysis following exposure to this drug. Similar changes in insertion abundance may reflect subtle differences in mutant fitness under the competitive growth conditions tested by TraDIS analysis^7^. Alternatively, a spontaneous suppressor mutation in the Δ*nhaA* deletion mutant could have restored the IPM resistance phenotype.

In conclusion, this study employed high-throughput, genome-wide antimicrobial-gene interaction profiling of MDR *K. pneumoniae* ST258 to generate an *in vitro* proof of concept that antimicrobial resistance can be reversed by targeting the SR. DedA was proven to be required for growth at therapeutic levels of CST, a key antibiotic for management of infections caused by this epidemic clone and other KPC-producing *K. pneumoniae* strains. The findings of the study call for more research to characterize the function of *dedA* and to assess whether this protein can be used as a target for developing helper drugs able to restore CST susceptibility in resistant strains. To date, the only antimicrobial helper drugs available in clinical practice are β-lactamase competitive inhibitors such as clavulanic acid and sulbactam, which are extensively used in combination with various penicillins (mainly amoxicillin, ampicillin and ticarcillin) to preserve their activity against bacterial strains that produce β-lactamase^49^. Similar therapeutic strategies based on the use of helper drugs deserve to be further explored to circumvent antimicrobial resistance in high-risk MDR clones such as *K. pneumoniae* ST258.

## Methods

### WGS of the model strain

The model strain of MDR *K. pneumoniae* ST258 (RH201207) was isolated from a wound infection in the UK. Whole genomic DNA was isolated using a phenol chloroform method and 2 μg was sequenced using the Pacific Biosciences (PacBio) RS II sequencing platform (Manlo Park, CA, USA). DNA was sheared to 20 kb and size selection was performed with Magbead (Pacific Biosciences), according to the manufacturer’s protocol. The library was sequenced using the P6 chemistry on 3 single-molecule real-time (SMRT) cells. Generated sequences were assembled *de novo* using SMRT analysis pipeline version 2.2.0 integrated with HGAP.3 (Pacific Biosciences) into 15 contigs. Illumina data (8 million 150 bp paired end reads) from the same genomic DNA sample was generated from a MiSeq (Illumina, USA) and was overlaid on these above contigs and 2 single nucleotide polymorphisms were identified and resolved using iCORN^50^.

Four plasmids were identified by *in silico* alignment to known replicons^51^ and BLASTn analysis against the non-redundant nucleotide NCBI database and consequently split into separate extrachromosomal DNA molecules from the chromosome; contigs per molecule were then annotated using PROKKA^52^ and finally submitted into the European Nucleotide Archive (ENA) under accession numbers for the chromosome and 4 plasmids LT216436-LT216440. Antibiotic resistance genes were identified using ResFinder^53^ and by manual inspection of known resistance genes.

### Media and reagents

Luria Bertani broth (LB) or agar (LA) were used for routine growth of bacteria. Cation-adjusted Mueller Hinton Broth II (MHB II) was used for antimicrobial susceptibility testing. SOC medium^54^ was prepared and used for the recovery of electroporated cells. All growth media were purchased from Becton Dickinson, USA. Tetracycline, aparamycin, hygromycin, CST sulphate, IPM, CIP, ethylenediaminetetraacetic acid (EDTA) and isopropyl-thiogalactopyranoside (IPTG) were purchased from Sigma-Aldrich, Denmark. Phusion hot start DNA polymerase kit was purchased from Thermo Fisher Scientific, USA. PCR primers were synthesized by TAG Copenhagen, Denmark. MasterPure Gram Positive DNA Purification Kit was purchased from Epicentre, USA, and sequencing library preparation kits were purchased from Illumina or PacBio, USA.

### TraDIS library construction

The tetracycline resistance cassette (Tet^R^) was amplified by PCR from pBR322^55^ (Table S1) using forward and reverse primers (Table S2) 5′ tagged with Tn*5* mosaic element and Phusion Hot Start DNA Polymerase Kit. PCR product was gel extracted using QIAquick gel extraction kit (Qiagen, Europe) and mixed with EZ-Tn*5* transposase (Epicenter, USA) and subsequently incubated at 37 °C for 30 min to prepare *in vitro* transposome complex, following manufacturer instructions. LB overnight culture of MDR *K. pneumoniae* ST258 was sub-cultured 1:100 in same fresh media supplemented with 0.7 mM EDTA^14^. Cells in early exponential growth phase were washed once with ice cold water and twice with 10% glycerol to make them electrocompetent, and finally re-suspended in 10% glycerol^54^. Transposase complex was electroporated into competent cells and recovered in SOC medium for 1 hour at 37 °C. Subsequently, mutants were selected on LA plate supplemented with 10 μg/ml of tetracycline. To construct >1 million mutant colonies, this procedure was repeated five times and multiple batches of colonies were collected and stored at −80 °C in 25% glycerol solution.

### TraDIS library antimicrobial exposure and sequencing

In duplicate, a 50 μl aliquot of the library, carrying approximately 5 × 10^7^ mutants, was diluted in 10 ml MHB II supplemented 2 μg/ml of antimicrobial or no antimicrobial (control). After 24 h of incubation at 37 °C, 100 μl of culture were transferred into 9.9 ml of fresh medium and incubated again for 24 h, and a 1 ml aliquot from each sub-culture was centrifuged at 10 000 rpm for 2 min. The resulting pellet was used for total DNA isolation using MasterPure Gram Positive DNA Purification Kit following manufacturer’s instructions. The quality of isolated DNA was evaluated using NanoDrop (Thermo Scientific, USA) and concentration of DNA was measured using Qubit dsDNA HS Assay Kit (Thermo Fisher Scientific, USA). For TraDIS sequencing, 2 μg of DNA was used for library construction following protocol described in the TraDIS Toolkit method^56^, using oligonucleotides (Table S2) specific for PCR enrichment of transposon insertion sites (DNA fragments tagged with transposon). Multiple PCR amplified fragment libraries were pooled and sequenced on a HiSeq2500 using an optimized TraDIS recipe^57^, yielding 32 million reads.

### TraDIS data analysis

Sequences containing the transposon tag were mapped against the RH201207 reference genome, using the Bio::TraDIS pipeline (https://github.com/sanger-pathogens/Bio-Tradis) to determine numbers of transposon insertions per gene. For each gene, an ‘insertion index’ was calculated so that the number of insertions in each gene was divided by total gene length, excluding 10% of the 3, end^56^. A histogram of insertion indices showed a bimodal distribution and gamma distributions were fitted to define the 2 nodes as the essential and non-essential genes. The log_2_-likelihood ratios for each gene were calculated, and considered essential if it was at least log_2_FC under the essential insertion index distribution, non-essential if it was at least log_2_FC likely to be under the non-essential insertion index distribution, as described previously in Dembek *et al*.^10^. Here, the essentiality cut-off was set at an insertion index of 0.0082 or and ambiguous gene fell between this and the non-essentiality cut-off of 0.013. Statistical analysis was performed in R. Read counts were normalized using the trimmed mean of M (TMM) method, and differences in insertions between antimicrobial exposed and unexposed samples were tested for statistical significance using the edgeR package version 3.4.2, as described previously^56^. We defined SRGs as genes with a log_2_FC change read counts of ≤−2 (≥4 fold change) at a False Discovery Rate (FDR) of 5%, which corresponds to q value ≤0.01. When multiple SRGs were placed on the same operon, the first structural gene was considered as representative of the entire operon in order to avoid redundancy from potential polar effects. The TraDIS sequencing data is published in the European Nucleotide Archive, under the accession numbers given in Table S3. Further, the read counts, mapping statistics and transposon prevalence information for each sample is also given in Table S3.

### Deletion of gene in MDR *K. pneumoniae* ST258

The genes identified by TraDIS were substituted individually by the Tet^R^ resistance cassette using lambda red recombinase tools^35^ that have been recently optimized for *K. pneumoniae* gene deletion^36^. First, a Tet^R^ cassette was cloned in the middle of two Flippase Recognition Targets (FRT) by substituting the chloramphenicol resistance cassette (Cam^R^) of pKD3-Cam^R35^ with Tet^R^ (Table S1). To swap the resistance markers, Tet^R^ cassette from pBR322 and the pKD3-Cam^R^ plasmid backbone, excluding Cam^R^ cassette, were amplified individually using two oligo pairs, oKD3-F/oKD3-R and otetR-F/otetR-R (Table S2), respectively, and fragments were ligated through blunt-end ligation using T4 ligase (Thermo Scientific, USA), resulting in the construction of pKD3-Tet^R^ (Table S1). For gene deletion, the Tet^R^ cassette was PCR amplified using primers (Table S2) attached with 59 bases homologous to the up- or down-stream sequence of the target gene and the resulting PCR product was purified by gel extraction. Temperature-sensitive lambda red recombinase plasmid (Table S1) was transformed into electrocompetent MDR *K. pneumoniae* ST258 and selected on LB plate containing 100 μg/ml hygromycin. The transformant was grown at 30 °C in LB supplemented with 0.7 mM EDTA and hygromycin. The recombinase genes were induced with 100 mM arabinose and induced cells were made electrocompetent. Purified Tet^R^ cassette was electroporated to competent cells and recovered at 37 °C for 2 hours. Finally, recombined mutant was selected on LA plate supplemented with 10 μg/ml tetracycline. Gene deletion was confirmed by PCR amplification and sequencing (Macrogen, Europe).

### Antimicrobial susceptibility testing of wild type and mutants

Antimicrobial susceptibility testing was performed by broth microdilution following CLSI standards^18^. MICs were recorded after 18 h of incubation at 37 °C.

### *In silico* homology study of proteins DedA

Bioinformatics analysis of DedA was performed following published analysis pipeline^58^. RH201207 annotated DedA protein sequence was taken from Artemis and sequence similarity with human protein was searched using NCBI Homo sapiens (human) Protein BLAST tools (http://blast.ncbi.nlm.nih.gov/Blast.cgi). Similarly, NCBI/BLAST/blastp suite tools were used to search sequence homology of DedA with protein sequences from different clinically relevant Enterobacteriaceae species. Four clinically relevant Enterobacteriaceae species (*Salmonella enterica, Shigella flexneri, Citrobacter freundii,* and *Escherichia coli*) were added in the analysis pipeline manually.

### Cloning of *K. pneumoniae dedA*

The *K. pneumoniae dedA* gene (Kpn*dedA*) was amplified from RH201207 genomic DNA using primers KdedA1 and KdedA2 (Table S2), digested with *SacI* and *HindIII* (New England Biolabs, UAS) and ligated with a similarly digested and dephosphorylated linearized fragment of vector pBAD-HisA (Invitrogen, USA) to construct plasmid pBAD-Kpn*dedA*. Since studied *K. pneumoniae* ST258 is resistant to ampicillin, the selection marker of both vector and clone was replaced by the apramycin resistance cassette of pIJ773. For this, both plasmids pBAD-HisA and pBAD-Kpn*dedA* were amplified using primers Bad1 and Bad2 (Table S2), digested with *XbaI* and dephosphorylated with Antartic phosphatase (New England Biolabs, USA) resulting in the linearized plasmid minus the Amp^R^ cassette. In parallel, the Apr^R^ cassette of pIJ773 was amplified using primers Apr1 and Apr2, digested with *XbaI* and ligated separately with the linearized plasmids described above using T4 ligase (Thermo Scientific, USA). Plasmids with Apa^R^ resistance marker were selected on LB agar plate supplemented with 50 μg/ml apramycin and KpnDedA protein was expressed using 0.002 to 0.1% arabinose inducer.

## Additional Information

**How to cite this article**: Jana, B. *et al*. The secondary resistome of multidrug-resistant *Klebsiella pneumoniae. Sci. Rep.* **7**, 42483; doi: 10.1038/srep42483 (2017).

**Publisher's note:** Springer Nature remains neutral with regard to jurisdictional claims in published maps and institutional affiliations.

## Supplementary Material

Supplementary Information

## Figures and Tables

**Table 1:** The colistin secondary resistome in *Klebsiella pneumoniae* ST258.

Gene/operon	Function	Colistin 2 μg/ml			Colistin 0.8 μg/ml		
		LogFC	LogCPM	q value	LogFC	LogCPM	q value
*dedA*	Putative integral membrane protein	−9.95	4.61	1.06E-48	−2.93	4.78	3.74E-25
*RH201207_02818*	Hypothetical protein	−9.67	4.35	3.83E-41			
*wabN*	Deacetylase, involved in lipopolysaccharide (LPS) biosynthesis	−9.33	6.34	4.77E-144	−4.77	6.39	5.84E-110
*galE*	UDP-galactose 4-epimerase, involve in LPS biosynthesis	−9.09	3.80	4.73E-30			
*wzabc*	Putative role in capsule polysaccharide (CPS) synthesis and outer membrane export of CPS	−8.52	6.90	1.09E-236			
***arnBCADTE***	Known resistance cassette, responsible for lipid A modification	−8.12	7.20	1.26E-292	−7.25	7.21	5.14E-295
*lpp*	Murein lipoprotein, plays role in membrane biogenesis and integrity maintenance	−8.03	2.83	1.17E-15			
*surA*	Periplasmic peptidylprolyl cis-trans isomerase chaperone, involved in correct folding and assembly of outer membrane proteins	−7.99	2.79	2.49E-13			
*RH201207_00408*	Putative glycosyltransferase involved in cell wall biosynthesis	−7.65	2.49	3.98E-12	−7.69	2.47	1.16E-11
*RH201207_00413*	Putative O-antigen ligase, involved in LPS biogenesis	−7.63	4.67	3.51E-48	−2.20	4.94	1.19E-17
*rnhA*	Ribonuclease H, endonuclease that specifically degrades the RNA of RNA-DNA hybrids	−6.71	1.72	1.34E-06			
*galU*	Have role in LPS biosynthesis and involved in virulence	−6.61	1.65	3.57E-06	−2.56	1.78	0.016688
*fabR*	Transcriptional repressor of cognate genes that involve unsaturated fatty acid biosynthesis	−6.55	5.91	8.49E-108			
*RH201207_00757*	Putative membrane protein	−6.22	7.97	0			
*Pgi*	Glucose-6-phosphate isomerase, housekeeping gene involved in membrane biosynthesis	−5.72	3.69	1.92E-23	−3.15	3.79	1.01E-14
*bamB*	Subunit of outer membrane protein assembly complex	−5.27	3.28	2.85E-16			
*tolQRAB*	Maintain envelope integrity	−5.24	3.25	7.52E-18			
*Pal*	Peptidoglycan-associated lipoprotein that contributes to serum resistance, maintains envelope integrity	−5.21	2.46	2.96E-10	−2.41	2.62	0.000517
*dedD*	Putative cell division associated protein	−5.12	4.30	8.89E-26			
*sdhD*	Putative succinate dehydrogenase complex subunit	−4.87	2.18	5.22E-08			
*hupA*	Transcriptional regulator HU subunit alpha	−4.36	2.49	1.85E-09			
*yhcB*	ubiquinol oxidase subunit III	−4.29	1.73	2.34E-05			
*RH201207_03912*	Putative glycosyltransferase, involved in LPS biogenesis	−4.18	9.40	0			
*gspA/rfbD*	Hypothetical protein	−4.17	9.81	0			
*fkpA*	FKBP-type peptidyl-prolyl cis-trans isomerase	−3.92	6.99	3.02E-114			
*degP*	Periplasmic serine endo-protease	−3.85	7.04	3.74E-138			
*RH201207_05041/42*	Hypothetical lipoprotein	−3.65	3.41	1.29E-12			
*tagA*	Hypothetical lipoprotein	−3.53	6.18	9.63E-66			
*cysC*	Putative adenylyl sulfate	−3.41	5.17	4.18E-30			
*ydgA*	Hypothetical protein	−3.36	6.31	1.09E-78			
*RH201207_01572*	Putative ECA polymerase	−3.26	4.08	1.58E-19			
*cpxR*	Response regulator receiver domain of envelope stress response two-component system	−3.06	3.23	7.80E-10			
*fre*	FMN reductase	−2.67	4.99	1.26E-17			
*mrcB*	penicillin binding protein 1b	−2.66	7.13	5.38E-114			
*miaA*	Putative tRNA delta(2)-isopentenyl-pyrophosphate transferase	−2.47	3.14	3.87E-07			
*envC*	Septal ring factor EnvC	−2.27	4.53	5.27E-11			

Genes/operons for which insertion sequence abundance was significantly (≥4 fold) depleted when the mutant library was cultured in the presence of 2 μg/ml or 0.8 μg/ml of colistin were identified by Transposon Directed Insertion-site Sequencing (TraDIS) analysis. Differences in insertion sequence abundance compared to the unexposed (control) culture of the library are expressed by log2 fold change (logFC). Number of insertion site sequence reads for the mentioned gene are presented by log2 counts per million (LogCPM). Quality of data is indicated by (q) value, where q ≤ 0.01 is significant. Known colistin resistance genes are presented in bold.

**Table 2:** The imipenem and ciprofloxacin secondary resistomes in *Klebsiella pneumoniae* ST258.

Gene/operon	Function	Imipenem			Ciprofloxacin		
		LogFC	LogCPM	q value	LogFC	LogCPM	q value
*nhaA*	pH dependent Na+/H+ antiporter	−2.22	4.51	2.38E-13			
*ydiE*	hypothetical protein				−2.67	2.20	0.013926

Genes/operons for which insertion sequence abundance was significantly (≥4 fold) depleted when the mutant library was cultured in the presence of 2 μg/ml of imipenem or ciprofloxacin were identified by Transposon Directed Insertion-site Sequencing (TraDIS) analysis. Differences in insertion sequence abundance compared to the unexposed (control) culture of the library are expressed by log2 fold change (logFC). Number of insertion site sequence reads for the mentioned gene are presented by log2 counts per million (LogCPM). Quality of data is indicated by (q) value, where q ≤ 0.01 is significant.
