# Supplementary material for: The secondary resistome of multidrug-resistant Klebsiella pneumoniae
Source: Sci Rep. 2017 Feb 15;7:42483. doi: 10.1038/srep42483 (PMC5309761; doi:10.1038/srep42483)

The secondary resistome of multidrug-resistant *Klebsiella pneumoniae*

Bimal Jana, Amy K Cain, William T Doerrler, Christine J Boinett, Maria C Fookes, Julian Parkhill and Luca Guardabassi

Table S1: List of strains and plasmids.

Strain name	Relevant geneotype	Source
RH201207	*Klebsiella pneumoniae* ST258	this study
RH201207Δ*dedA*	DedA gene is substituted with Tet^R	this study
RH201207Δ*nhaA*	NhaA gene is substituted with Tet^R	this study
**Plasmids**		
pBR322	pBR322 Amp^R Tet^R	Ref 51
pACBSR-Hyg^R	Carrying arabinose-inducible λ-Red recombinase genes and hygromycin resistance cassette	Ref 35
pKD3-Cam^R	Chloramphenicol resistance cassette (*cat)* is placed in the middle of two Flippase Recognition Targets (FRT)	Ref 34
pKD3-Tet^R	Original *cat* gene of pKD3 was replaced by tetracycline resistance gene (*tetA)*	this study
pIJJ773	Plasmid carrying apramycin resistance cassette (Apr^R) in the middle of two Flippase Recognition Targets (FRT)	Ref 35
pBAD-HisA	Expression vector; *araBAD* promoter, Amp^R	Invitrogen
pBAD-Kpn*dedA*	*Klebsiella pneumoniae dedA* gene was cloned in pBAD-HisA vector under *araBAD* promoter, Amp^R	this study
pBAD-Apr^R	Original Amp^R cassette of pBAD-HisA vector was substituted by apramycin resistance gene (Apr^R) of plasmid pIJJ773	this study
pBAD-Apr^R-Kpn*dedA*	*Klebsiella pneumoniae dedA* gene was cloned under arabinose promoter in pBAD-Apr^R vector	this study

Table S2: List of primers.

**Primers to amplify Tn element: mosaic element tagged Tet^{R} cassette[a]**	
Forward primer	5'Po4-CTGTCTCTTATACACATCTCAACCATCAATGTTTGACAGCTTATCATC
Reverse primer	5'Po4-CTGTCTCTTATACACATCTCAACCCTGATCAGGTCGAGGTGGCCCG
**Primers to amplify Tn insertion sites[b]**	
Amplify 5' Tn attached site	5'AATGATACGGCGACCACCGAGATCTACACACTGTGATAAACTACCGCATTAAAGCTTATCG
Amplify 3' Tn attached site	5'AATGATACGGCGACCACCGAGATCTACACTTGCGTCGCGGTGCATGGAGCC
**Primers for HiSeq sequencing**	
5' reading primer	5'CGATGATAAGCTGTCAAACATTGATGGTTGAGATGTGTA
3' reading primer	5'CACCTCGACCTGATCAGGGTTGAGATGTGTA
**Primers to knock out the gene; amplify Tet^{R} cassette in the middle of two FRT sequences that are attached with up or down streams sequences of target gene[c]**	
Forward primer_*nhaA*	5'CTGATTTTTTCGAGTTATCAGTATGTGTATTATGCTTTTATCGAGTTATTCACCAACAGGATGTTTGACAGCTTATCATC
Reverse primer_*nhaA*	5'TTATACCGCGAGGCGAGTATCCGTCACGCGCTGACGCAGTATGAGGTAGCCGACGACCGCTCAGGTCGAGGTGGCCC
Forward primer_*dedA*	5'TAGGGTCATTAAGGGTAAAGACAAACATGGACTTGATTCACTTTTTAATTGATTTCATCCGTGTAGGCTGGAGCTGCTTCG
Reverse primer_*dedA*	5'AAAAAGTGGTCAAACCGCTGCGCGTGACGCTCTGCACCGCCACGCGCAACACAGGCTAAAATGGGAATTAGCCATGGTCC

**Sequencing primer to confirm the knock out**	
Seq_Forward *_nhaA*	5'CCTTTGTTGATATCCGGCCCG
Seq_Reverse *_nhaA*	5'CTGCGGCGTCAGAAACAGC
Seq_Forward *_dedA*	5'TGCTGGCAGCAAAAGACAGG
Seq_Forward *_dedA*	5'CGATGCCAGTTTTG CGTAAGG
**Primer associated with gene cloning**	
KdedA1	5'GTTGGAGCTCATGGACTTGATTCACTTTTTAATTG (*SacI* site underlined)
KdedA2	5'GGCCAAGCTTCTACTTCGCCTGCTTCGC (*HindIII* site underlined)
BAD1	5'GCTCTAGACTGTCAGACCAAGTTTACTC (*XbaI* site underlined)
BAD2	5'GCTCTAGAACTCTTCCTTTTTCAATATTATTGAAG (*XbaI* site underlined)
Apr1	5'GCTCTAGAATGTCATCAGCGGTGGAGTG (*XbaI* site underlined)
Apr2	5'GCTCTAGATCAGCCAATCGACTGGCGAG (*XbaI* site underlined)
oKD3-F	5'TTTTCATCTTCCGTCACAGGTAGG
oKD3-R	5'TTTTCTTCCTATTCCGAAGTTCC
otetR-F	5'GTGTAGGCTGGAGCTGCTTCG
otetR-R	5'ATGGGAATTAGCCATGGTCC

[a] Underlined parts are mosaic elements.

[b] Underlined parts are adaptor sequences.

[c] Underlined parts complement with Tet^{R} cassette.

Table S3: ENA accession number of raw data files.

Sample	ENA Accession no	Total Reads	% Tn	% Mapped	Unique Insertion Sites	Bp/ Insertion
207_LB_1	ERS651624	2611326	97.1	81.1	341493	17.2
207_LB_2	ERS651625	2091291	97.0	80.8	317243	18.5
207_cip_1	ERS651626	2067508	97.1	81.6	317323	18.5
207_cip_2	ERS651627	1595945	97.3	81.9	288590	20.3
207_imp_1	ERS651628	1855613	97.4	83.3	304482	19.3
207_imp_2	ERS651629	1992086	97.1	83.1	314594	18.7
207_1_4_col_1	ERS651636	2073789	97.6	83.5	254158	23.1
207_1_4_col_2	ERS651637	2135957	97.3	83.0	256770	22.9
207_1_10_col_1	ERS651638	2299395	97.7	82.2	329553	17.8
207_1_10_col_2	ERS651639	2193574	97.9	82.6	329344	17.8

Fig. S1: *Confirmation of dedA gene deletion by whole genome sequencing of RH201207ΔdedA mutant*. Alignment of shotgun sequence fragments of RH201207 Δ*dedA* with the chromosomal sequence of the wild type RH201207 using Artemis software confirmed the absence of *dedA*. Nucleotide positions in the chromosome of RH201207 are indicated at the bottom.

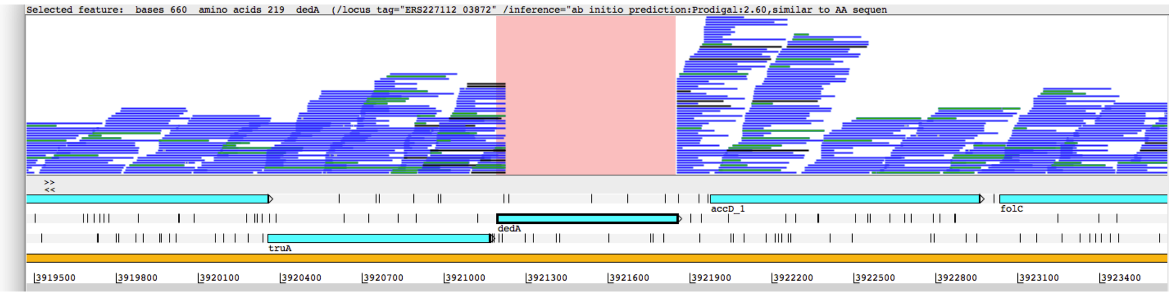

dedA

Fig. S2: *Complementation of DedA reintroduced colistin resistance in RH201207ΔdedA mutant*. *dedA* gene was cloned under arabinose promoter of pBAD-Apr^R vector and transformed to RH201207 wild type and RH201207Δ*dedA* mutant (labeled Kpn and Δ*dedA*, respectively). 10 fold serially diluted culture of wild type and mutant, carrying empty pBAD-Apr^R vector (-) or cloned *dedA* (+), were spotted on agar plate supplemented with 50 µg/ml apramycin , 0.1% arabinose and 0, 1.5 or 8 µg/ml of colistin. Growth of bacterial spots was recorded after 24 hours of incubation at 37°C.

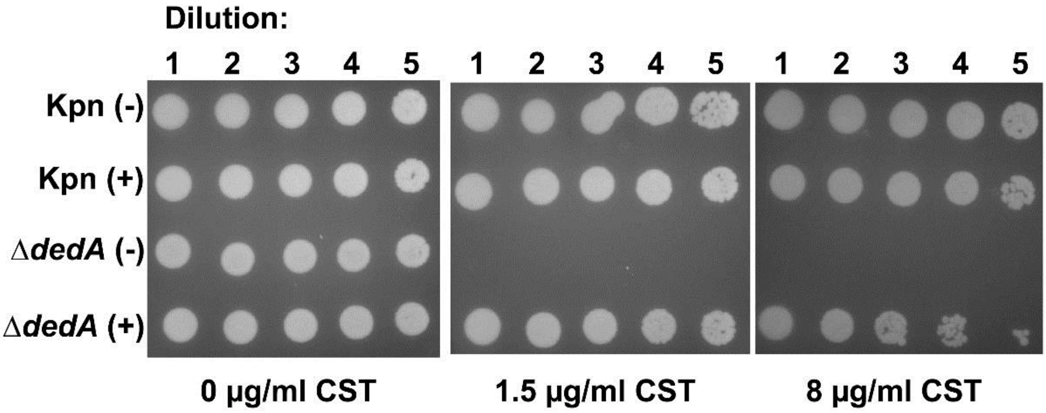

Fig. S3: *Growth of RH201207 and dedA mutant*. Overnight cultures were sub-clultured in fresh MHB II media and grown for 8 hours at 37°C with continuous shaking. OD_{600} of cultures were taken at every hour and plotted over time.

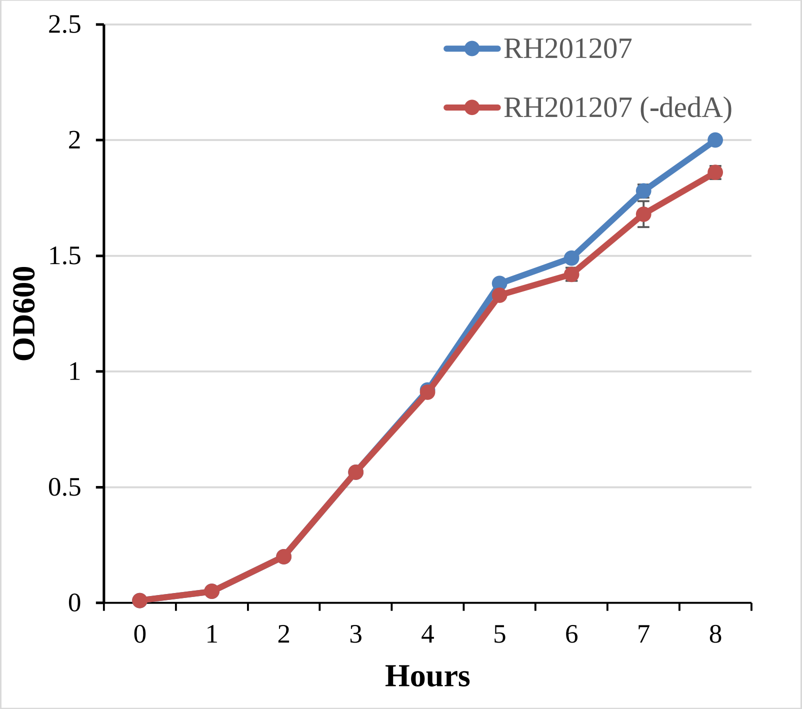

Supplement: Supplementary Information [file srep42483-s1.pdf]
